# Nanocomposite Based on Functionalized Gold Nanoparticles and Sulfonated Poly(ether ether ketone) Membranes: Synthesis and Characterization

**DOI:** 10.3390/ma10030258

**Published:** 2017-03-03

**Authors:** Iole Venditti, Laura Fontana, Francesca A. Scaramuzzo, Maria Vittoria Russo, Chiara Battocchio, Laura Carlini, Laurent Gonon, Vincent H. Mareau, Ilaria Fratoddi

**Affiliations:** 1 Department of Chemistry, Sapienza University of Rome, P.le A. Moro 5, 00185 Rome, Italy; iole.venditti@uniroma1.it (I.V.); laura.fontana@uniroma1.it (L.F.); mariavittoria.russo@uniroma1.it (M.V.R.); 2 Department of Basic and Applied Sciences for Engineering, Sapienza University of Rome, Via A. Scarpa 14, 00161 Rome, Italy; Francesca.scaramuzzo@uniroma1.it; 3 Department of Sciences, and CISDiC, Roma Tre University, Via della Vasca Navale 79, 00146 Roma, Italy; chiara.battocchio@uniroma3.it (C.B.); Laura.carlini@uniroma3.it (L.C.); 4 CNRS, CEA, INAC-SPrAM, University Grenoble Alpes, F-38000 Grenoble, France; laurent.gonon@ujf-grenoble.fr (L.G.); vincent.mareau@ujf-grenoble.fr (V.H.M.)

**Keywords:** gold nanoparticles, sulfonated poly(ether ether ketone) membranes, metal nanoparticles, proton exchange membranes, atomic force microscopy, fluid cell

## Abstract

Gold nanoparticles, capped by 3-mercapto propane sulfonate (Au-3MPS), were synthesized inside a swollen sulfonated poly(ether ether ketone) membrane (sPEEK). The formation of the Au-3MPS nanoparticles in the swollen sPEEK membrane was observed by spectroscopic and microscopic techniques. The nanocomposite containing the gold nanoparticles grown in the sPEEK membrane, showed the plasmon resonance λ_max_ at about 520 nm, which remained stable over a testing period of three months. The size distribution of the nanoparticles was assessed, and the sPEEK membrane roughness, both before and after the synthesis of nanoparticles, was studied by AFM. The XPS measurements confirm Au-3MPS formation in the sPEEK membrane. Moreover, AFM experiments recorded in fluid allowed the production of images of the Au-3MPS@sPEEK composite in water at different pH levels, achieving a better understanding of the membrane behavior in a water environment; the dynamic hydration process of the Au-3MPS@sPEEK membrane was investigated. These preliminary results suggest that the newly developed nanocomposite membranes could be promising materials for fuel cell applications.

## 1. Introduction

In recent years, the preparation of composite materials has been one of the growing demands for energy conversion and storage applications [1,2,3]. Among others, metal nanoparticles dispersed in polymeric or oxide matrices are envisaged as promising candidates for catalysts or sensor interactive materials [4,5,6]. In fact, the nanoscale provides a high specific surface area, favoring a better dispersion of metal nanoparticles and an increase in their intrinsic activity [7,8]. In particular, metal nanoparticles, and especially gold nanoparticles, combine the synthetic versatility of surface functionalization [9,10,11] with their inherent ability to act as catalysts or carriers, allowing their use in a variety of applications, ranging from plasmonics, sensors, and energy applications [12,13], to well assessed studies in biotechnology and nanomedicine [14,15,16,17].

A growing amount of attention in the literature has been paid to the use of nanocomposite membranes for energy applications [18,19]. The development of polymeric membranes has rapidly grown in recent years [20], with the general goal of obtaining tailored physical and transport properties for a variety of applications, with a strong interest in nanostructured features [21,22]. The improvement of mechanical properties is one of the most important objectives that can be fulfilled by introducing a filler [23]. Nanoparticles are particularly interesting thanks to their easy surface functionalization and high surface/volume ratio [24]. Hydrophobic and hydrophilic metal nanoparticles have been prepared and a fine control of size, surface chemistry, and assembly has been achieved [25,26]. Several methods for the deposition of nanoparticles (NPs) in a host membrane have been developed over the past few decades, such as atomic layer deposition [27], an intermatrix synthesis (IMS) technique [28], and an incorporation [29,30] and in situ sol-gel method [31]. All of these composite systems have the aim of controlling the nanoparticles and membrane morphologies to prevent NP agglomeration and corrosion, and to enhance their specific properties [32,33].

The poly(perfluorosulfonic) acid membrane (Nafion-117) has received much attention as a host membrane for a variety of nanoparticles, i.e., Pt, Au, Ag, and bimetallic nanoparticles [34,35]. The interest in this type of composite membrane is due to the combination of the specific properties of the NP and the polymer matrix, allowing a wide range of applications (fuel cells, sensors, and actuators [36,37]). Nafion-117 represents one of the most used membranes in fuel cell applications. However, its high cost and ecological impact, as well as its limited thermomechanical properties [38], stimulated the research towards alternative polymeric systems. Among others, sulfonated poly (etheretherketone) (sPEEK) membranes have been integrated into functional devices [39], thanks to their good solvent resistance, high thermal stability, and excellent mechanical properties due to the aromatic backbone. Sulfonated poly(etheretherketone) (sPEEK) represents a low cost alternative [40] to Nafion-117 and takes advantage of the easy post-modification procedures for the introduction of sulfonate end groups (–SO_3_H) [41,42]. Sulfonation can, in fact, be easily carried out by using sulfuric acid on polymeric PEEK membranes [43]. Depending on the sulfonation degree, the proton conductivity, the thermo-mechanical stability of sPEEK membranes can be tailored to fulfill the requirements. In general, the hydrophilicity and proton conductivity [44] of the sPEEK membrane increases with its extent of sulfonation, due to the well-assessed aggregation phenomena of sulfonic functional groups that give rise to hydrophilic domains where the mobility of protonic charges is optimized.

Several studies have demonstrated that a good membrane hydrophilicity and proton conductivity are reached for a sulfonation degree in the range of 60%–80% and water uptake between 20% and 40%, with a conservation of the structural and mechanical robustness of the PEEK membranes [45,46]. Low sulfonated PEEK membranes show a high thermal and chemical stability, but, unfortunately, a relatively low proton conductivity [47]. To solve this key issue, sPEEK membranes were used as a matrix for hybrid/blend membranes. In this context, sPEEK membranes, decorated or imbedded with nanofillers, were obtained [48,49] by impregnation and intercalation methods [50,51,52].

The combination of the easy processability of an organic polymer and the improved mechanical and optical properties of NPs, can also be fundamental for the enhancement of the selectivity and permeability of membranes [53], and considering this, we chose to use hydrophilic metal nanoparticles. In fact, hydrophilic gold nanoparticles show a high stability during the colloidal phase thanks to surface ligands and open new perspectives for their use as fillers in membrane applications. In this work, the development of an efficient procedure for the inclusion of hydrophilic gold nanoparticles in sPEEK polymer membranes, is presented. Gold nanoparticles functionalized with 3-mercapto propane sulfonate (3MPS) are considered here as a model system; a proof of concept to be extended in the future in order to optimize not only the hydration degree, but also the chemical stability and the protonic conductivity of the proton exchange membrane for fuel cell applications.

## 2. Results and Discussion

### 2.1. Au-3MPS@sPEEK Nanocomposite by In Situ Synthesis

**Figure 1:**
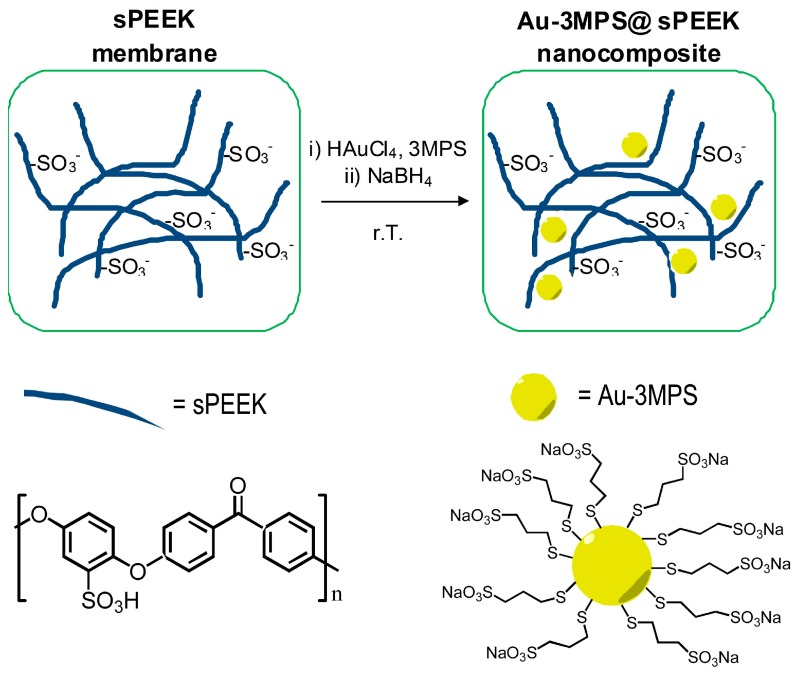
Schematization of the Au-3MPS@sPEEK nanocomposite formation.

The synthesis of the Au-3MPS@sPEEK nanocomposite was carried out by the impregnation of the Au(III) precursor and 3MPS stabilizing thiol on reacidified and hydrothermally treated sPEEK membranes. This treatment, applied on the pristine sPEEK membrane, allows the production of a host sPEEK membrane with well-defined and swell hydrophilic domains, suitable for hosting the precursors [54]. By adding the strong reducing agent NaBH_4_ to the reaction mixture, after 30 min of impregnation, a rapid color variation was observed, from yellow to brown, and the white translucent sPEEK membranes gained a brown color upon the sorption and reduction process, remaining unchanged upon rinsing. Isolated Au-3MPS nanoparticles were recovered from the reaction solution mixture, demonstrating that the host sPEEK membrane impregnation was not completed before NaBH_4_ addition. The immobilization process of Au-3MPS nanoparticles in the sPEEK matrix is schematized in Figure 1; the nanocomposite showed stability over a testing period of three months.

**Figure 2:**
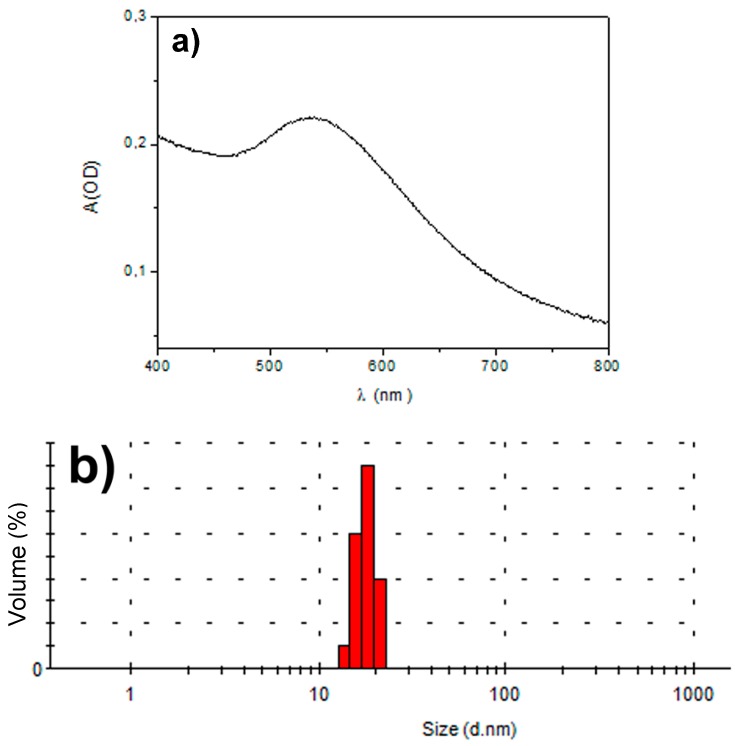
Characterization of Au-3MPS nanoparticles in water: (**a**) UV-vis spectrum; (**b**) DLS data.

The formation of Au-3MPS nanoparticles recovered from the reaction mixture was confirmed using UV–visible spectral analysis (Figure 2a). The characteristic absorption peak was observed at 540 nm, due to the surface plasmon resonance of AuNPs. The DLS results showed that AuNPs have a narrow size distribution with an average size of about 14 ± 3 nm (Figure 2b).

**Figure 3:**
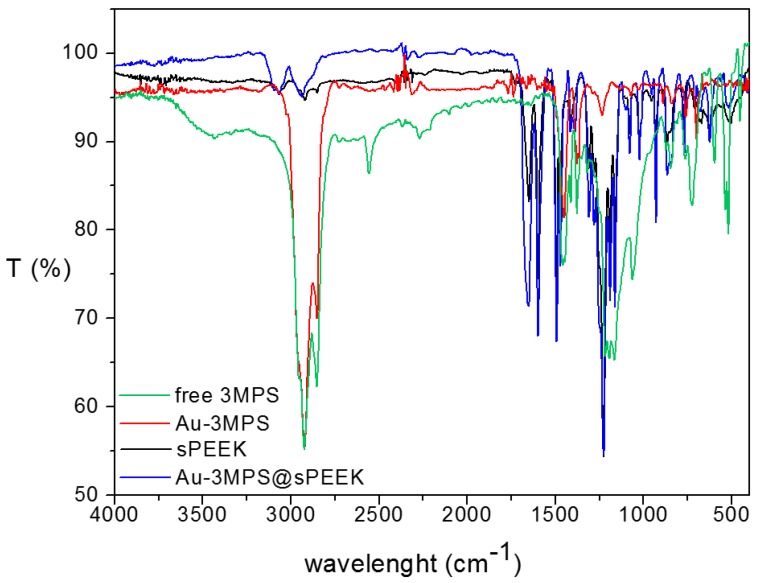
FTIR Transmission spectra of the nanocomposite Au-3MPS@sPEEK (blue line) compared with the sPEEK host membrane (black line), Au-3MPS NPs collected from the impregnation solution (red line), and free 3MPS thiol (green line).

The Au-3MPS@sPEEK nanocomposite membrane was then characterized using Transmission FTIR, XPS, and AFM techniques, and was compared with the native host sPEEK membranes and Au-3MPS and free 3MPS samples. In the FTIR spectra (Figure 3 and Supporting Information section), the absorption peaks at 1188 and 1654 cm^−1^ correspond to the –Ar–O–Ar– and –Ar–C(=O)–Ar– groups, respectively, indicating that the structure of ether ketone is evident in both the sPEEK and Au-3MPS@sPEEK samples. The absorption peak of the benzene ring at 1595 and 1472 cm^−1^ was observed, together with the Ar–O–Ar and O=S=O bands at 1225 cm^−1^, due to the asymmetric stretching vibration peak [55]. In the Au-3MPS@sPEEK nanocomposite, only a few shifts are envisaged with respect to the dominant sPEEK bands, together with some characteristic stretching modes (at 1384, 1160, and 1050 cm^−1^), due to the 3MPS ligand. Moreover, the absence of the stretching mode S-H in the Au-3MPS@sPEEK sample, at about 2550 cm^−1^, indicates the absence of free thiol 3MPS. Therefore, the 3MPS observed in the Au-3MPS@sPEEK nanocomposite membrane corresponds to Au-3MPS in the membrane, and not trapped free 3MPS.

**Figure 4:**
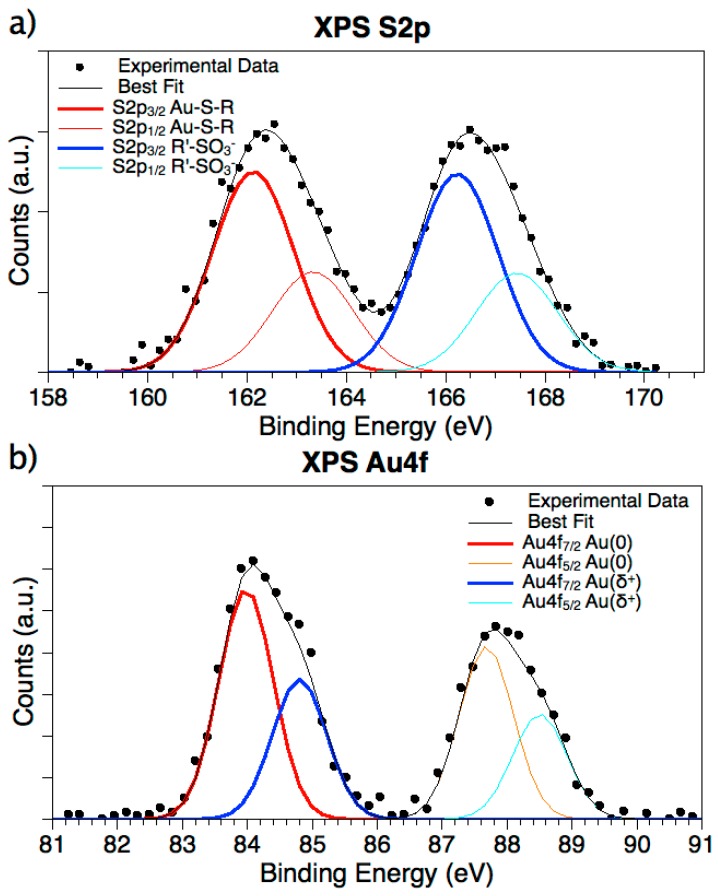
XPS spectra of Au-3MPS@sPEEK. (**a**) XPS S2p spectrum; two signals (each one composed by two spin orbit components: S2p_3/2_ and S2p_1/2_) associated with S atoms bonded to gold atoms at the NP surface (S2p_3/2_ red, S2p_1/2_ orange), and to sulphonate moieties (S2p_3/2_ blue, S2p_1/2_ cyan), are observed; (**b**) Au4f spectrum; two signals (each one composed by two spin orbit components: Au4f_7/2_ and Au4f_5/2_) associated with metallic gold atoms at the bulk of NPs (Au4f_7/2_ red, Au4f_5/2_ orange), and Au atoms bonded to sPEEK through sulfur (Au4f_7/2_ blue, Au4f_5/2_ cyan), are observed.

X-ray photoemission spectroscopy measurements were performed on Au-3MPS@sPEEK nanocomposite membranes, in order to ascertain the successful binding of 3MPS to gold NPs in the membrane framework. A complete collection of XPS data, i.e., BE, FWHM, atomic ratios, and assignments, is reported in the supplementary information. XPS data collected at S2p and Au4f core levels confirmed the presence of AuNPs stabilized with covalently bonded 3MPS, at least in the first layers of the membrane (XPS sampling depth is of some nanometers), and the absence of free thiol, as already observed by the FTIR measurements (analysis characteristic of the bulk of the membrane). As reported in Figure 4a, the S2p spectrum of Au-3MPS@sPEEK shows two spin-orbit pairs, indicative of S atoms in two different chemical states. The first signal (S2p_3/2_, BE = 162.13 eV, BE: binding energy) is associated with sulphur atoms covalently bonded to gold atoms of the AuNP’s surface [56]; the signal at higher BE values (S2p_3/2_ BE = 166.24 eV) is consistent with the sulfonate moiety. The intensity ratio between the two signals is 1/1, as expected by the proposed molecular structure. Free 3MPS molecules would be expected to give rise to a third S2p signal with the main S2p_3/2_ spin-orbit component at about 164 eV, typical of free thiol end groups (free or physisorbed molecules). Since this signal is not observed, we can affirm with reasonable confidence that all 3MPS molecules are chemically bonded to the gold NPs. Au4f data confirmed the chemical interaction between AuNPs and ligands; as shown in Figure 4b, the Au4f spectrum shows different components, with a main pair of spin-orbit components associated with metallic gold atoms in the bulk of nanoparticles (Au4f7/2 BE = 83.98 eV), and a less intense signal at higher BE values, indicative of partially oxidized gold atoms at the NPs surface, covalently bonded to the thiol moiety of 3MPS [57,58].

### 2.2. AFM Studies

The characterization reported in the literature on sPEEK and sPEEK decorated with nanofillers, has mainly been performed via XPS, XRD, or EDX [59,60,61] to investigate the composition, and by FTIR, SEM, and TEM, to provide detailed information on the membrane surface and the inner section [62,63]. The surface roughness is usually measured using profilometers [64,65], while few groups have performed AFM [66,67,68,69] characterization, despite the well-established use of this technique to characterize multi-layer systems, compare the roughness upon the addition of subsequent charges [70], and check the homogeneous distribution of the nanofillers within the membrane.

**Figure 5:**
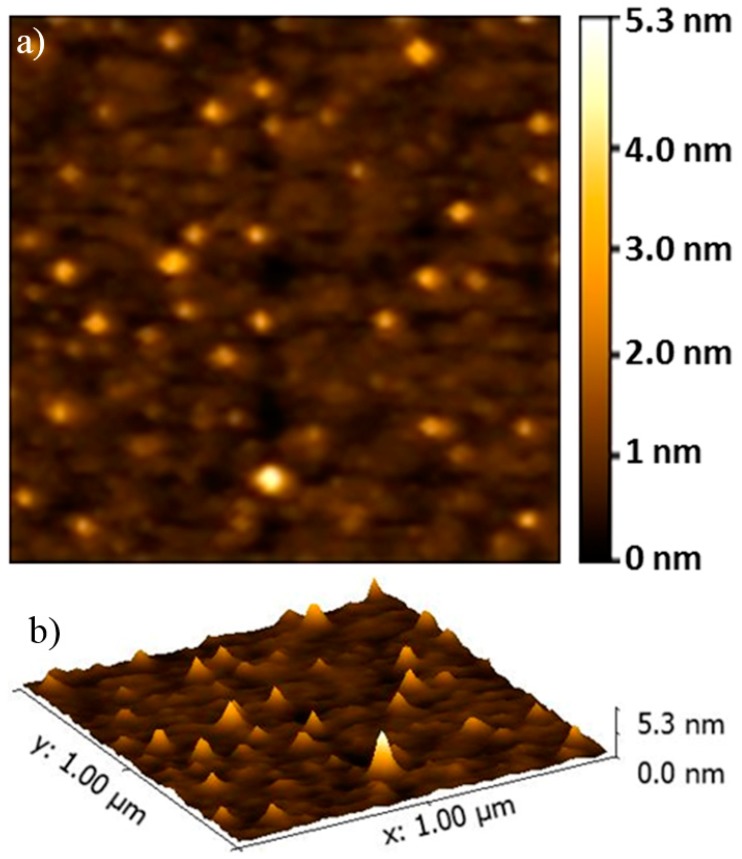
AFM images of Au-3MPS deposited on Si/SiO_2_: 1 μm × 1 μm 2D (**a**) and 3D (**b**) topography.

Both Au-3MPS nanoparticles and Au-3MPS@sPEEK nanocomposite membranes were subjected to AFM analysis, to observe their morphology and surface roughness. As a first experiment, Au-3MPS nanoparticles deposited on a Si/SiO_2_ substrate were characterized and a typical image is presented in Figure 5. 

**Figure 6:**
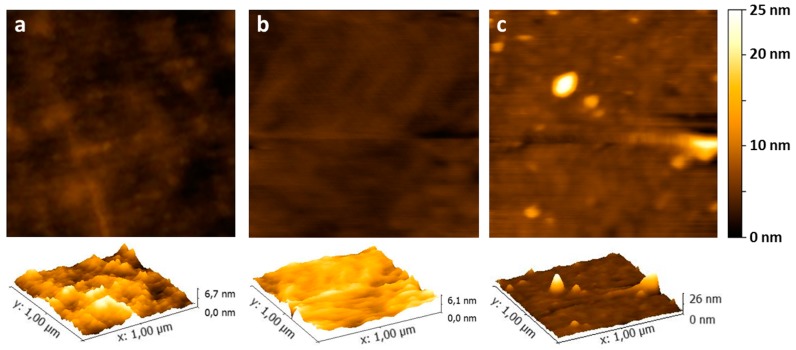
2D and 3D AFM images of the 1 μm × 1 μm area of the pristine sPEEK membrane (**a**); the dry sPEEK membrane after reacidification and hydrothermal treatment (**b**) and the membrane with Au-3MPS (**c**).

The Au-3MPS NPs show a regular round shape, with a maximum z-dimension (height) of about 5 nm. Considering the curvature radius of the tip used here (about 8 nm), only the z-dimension of the observed NPs is representative of their real dimensions. Their lateral dimensions are enlarged by the tip dimensions (always the smaller object taking an image of the larger one), by 5 nm for the NPs height, and therefore, the real diameter is smaller than that found by DLS measurements, because in the latter case, the solvent plays a role in the swelling of nanoparticles. In order to obtain a complete morphological characterization, the pristine dried sPEEK membrane has been compared with the sPEEK membrane after hydrothermal treatment and the AuNP’s embedded membrane. Figure 6a shows the 2D and 3D topography AFM images of the pristine sPEEK membrane in air. As it is possible to view, the pristine membrane appears extremely flat, without a special texture, and has a very low average roughness value, i.e., 0.82 nm evaluated on a 1 μm × 1 μm area. The reacidification and hydrothermal treatment does not seem to affect the membrane: for dried samples, the aspect remains almost unaltered (Figure 6b), while the change in the average roughness value (now 0.50 nm on a 1 μm × 1 μm area) is negligible. Figure 6c shows the 2D and 3D topography AFM images in air, obtained upon membrane embedding with NP and subsequent drying. The pictures clearly show that, even though the roughness remains nearly unaltered (i.e., 0.98 nm evaluated on a 1 μm × 1 μm area), the Au-3MPS@sPEEK membrane impregnated with Au-3MPS has quite a different aspect when compared to those already discussed. In particular, the Au-3MPS@sPEEK composite membrane is generally characterized by the presence of objects with dimensions up to 30 nm on Z-axes, which are supposed to be aggregates of nanoparticles [71]. In order to achieve information on the presence of Au-3MPS in the membrane, FESEM images have been acquired and a cross sectional view is reported in the Supporting Information Section. AuNPs have been observed in the internal sections and confirmed by EDX analysis, allowing us to assess their presence in the membrane.

**Figure 7:**
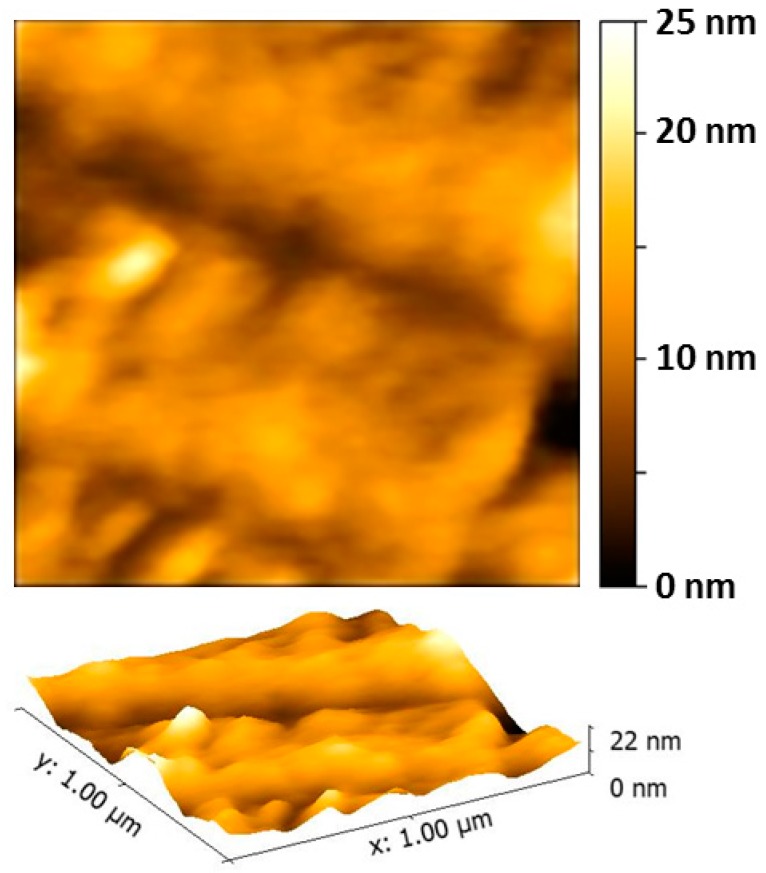
2D and 3D AFM images in fluid of the 2.5 μm × 2.5 μm area of the Au-3MPS@sPEEK membrane.

In order to study the behavior of the membrane in wet condition, AFM measurements were then carried out in fluid. As is well known, treatment with H_2_O demonstrates the sPEEK membrane hydrophilicity that requires fluid cell periodic refilling. AFM measurements in liquid have been performed at both t = 0 and t = 1 h, after refilling the cell. Pictures collected just after depositing one drop of water, show the tendency of the membrane to absorb the solvent. The main effect of the wetting is undoubtedly the hydration of the membrane, (Figure 7) whose roughness increases up to 4.98 nm on a 1 μm × 1 μm area. Along with the hydration of the membrane, some previously observed features disappeared, such as the presence of nanoparticles of the order of 10 nm, observed for the dry membrane.

**Figure 8:**
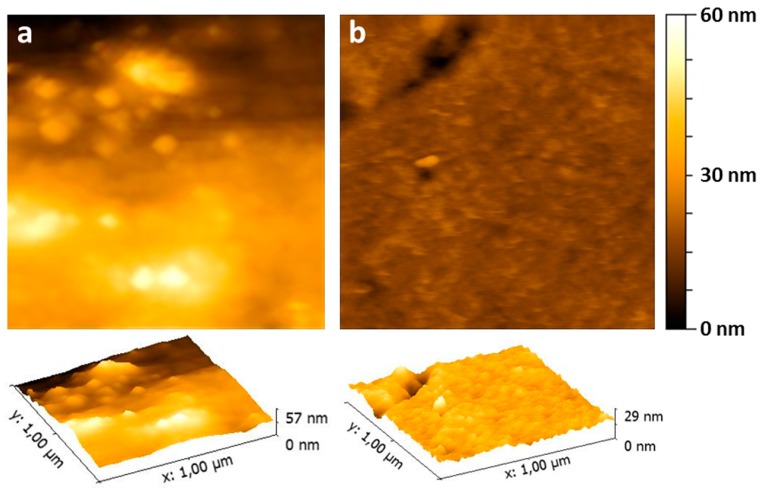
2D and 3D AFM images of the 1 μm × 1 μm area of the Au-3MPS@sPEEK membrane wet for deposition of one drop of water and dried (**a**); and 1 h after drying (**b**), which show the reversibility of the process.

Finally, the effect of pH on the Au-3MPS@sPEEK membrane has been tested, by dipping the samples at pH = 5 and pH = 2. The measurements at different pH levels showed no particular differences. It is certain that the hydration of the membrane is not instantaneous, and that it is reversible. In fact, after keeping the membrane wet for 1 h upon the deposition of one drop, drying, and measuring its roughness, a maximum height of 113 nm was reached, with a roughness of 8.98 nm, evaluated on a 1 μm × 1 μm area. On the other hand, 1 h after drying, it is possible to observe a partial reduction of the roughness, with a value of 1.49 nm (Figure 8).

## 3. Materials and Methods

### 3.1. Materials

All reagents and analytical grade solvents were purchased from commercial sources and used as received, unless otherwise stated: chloroform, ethanol, methanol, hydrochloric acid, sulfuric acid, tetrachloroauric(III) acid trihydrate (HAuCl_4_·3H_2_O), sodium borohydride (NaBH_4_), and 3-mercapto propane sulfonate (3MPS). Poly(ether ether ketone) (sPEEK) commercial membranes were purchased from Fumatech^®^ (E-750, Bietigheim-Bissingen, Germany) with an ionic exchange capacity (IEC) of 1.33 meq·g^−1^ (milli molar SO_3_H per gram of polymer) and a membrane thickness of about 30 µm. Reduction reactions of Au(III) to AuNPs were carried out under Argon in deionized water.

Prior to the AuNPs synthesis, the host sPEEK membrane was prepared as follows: a strip of polymer was divided into squares of approximately 2 cm. The polymeric membranes were immersed in a solution of H_2_SO_4_ (96% Aldrich, Milano, Italy) (1 M) for 4 h at 60 °C under stirring, while maintaining the vessel in the dark. Then, membranes were kept at 80 °C for three days, in deionized water. After that, the membranes were washed three times with 100 mL of deionized H_2_O and were ready to be used.

### 3.2. Preparation of Au-3MPS@sPEEK Nanocomposite Membranes

The whole process was performed in the dark, to avoid any photochemical modification due to the chromophore groups of sPEEK. After the preparation (reacidification and hydrothermal treatment) of sPEEK membranes, as reported above, the host sPEEK samples were used as a template for the synthesis of the Au-3MPS@sPEEK composite, with an Au/3MPS molar ratio equal to 1/4. In the following paragraph, the procedure is described. sPEEK membranes (squares of 2 cm, thickness 30 microns) were put in a two-neck flask for precursor impregnation in the host sPEEK membrane, in the presence of deionized water as a solvent (5 mL), for 1 h. HAuCl_4_ (100 mg in 10 mL of deionized water) was added, together with 3MPS (169 mg in 10 mL of deionized water), at room temperature, by following the procedure employed in the literature [72]. After stirring for 30 min, a solution of the reducing agent NaBH_4_ (94.5 mg in 5 mL was added) and the reaction mixture, was allowed to react at room temperature. The solution rapidly changed from yellow to dark brown, and after 2 h of continuous stirring, the brown membranes were accurately rinsed with 100 mL of deionized water and immersed for 30 min, to remove adsorbed nanoparticles. The Au-3MPS@sPEEK nanocomposite membranes were dried and then analyzed by AFM. The reaction mixtures containing dispersed AuNPs, after removal of the membrane and purification through three centrifuges in deionized water (5000 rpm for 10 min), were analyzed by UV-vis spectroscopy and DLS measurements, to measure the dimensions of the Au-3MPS NPs grown in solution.

### 3.3. Characterization Methods

Transmission FTIR spectra have been recorded from deposited films by casting from the DMF solution, by mixing the sample with solvent at room temperature for 1 h, using KRS-5 cells, with a Bruker Vertex 70 spectrophotometer (Bruker, Milano, Italy). UV-vis spectra were run in H_2_O solution by using quartz cells with a Varian Cary 100 Scan UV-vis spectrophotometer (Varian, Milano, Italy). The size and size distribution of Au-3MPS in H_2_O solution have been investigated by means of a dynamic light scattering (DLS) technique, by using a Brookhaven instrument (Brookhaven, Holtsville, NY, USA) equipped with a 10 mW HeNe laser at a 632.8 nm wavelength, at a temperature of 25.0 ± 0.2 °C [73]. Correlation data have been acquired and fitted in analogy to our previous work. X-ray photoelectron (XPS, home-made instrument) spectra were recorded using a custom designed spectrometer, described in previous studies [74] and equipped with a non-monochromatized Mg Kα X-ray source (1253.6 eV pass energy 25 eV, step 0.1 eV). The spectra have been acquired on sPEEK membranes and Au-3MPS@sPEEK samples. The spectra were energy referenced to the C1s signal of aliphatic C atoms, which had a binding energy BE pf 285.00 eV. Atomic ratios were calculated from peak intensities using Scofield's cross-section values and calculated λ factors were used [75]. Curve-fitting analyses of C1s, S2p, Cl2p, B1s, O1s, and Au 4f spectra were performed, using Gaussian profiles as fitting functions, after the subtraction of a Shirley-type background. For quantitative data, the BE values were referred to the NIST database [76].

S2p_3/2,1/2_ and Au4f_7/2,5/2_ doublets were fitted by using the same full width at half-maximum (FWHM) for each pair of components of the same core level, a spin–orbit splitting of, respectively, 1.2 eV and 3,7 eV, and branching ratios of S2p_3/2_/S2p_1/2_ = 2/1 and Au4f_7/2_/Au4f_5/2_ = 4/3. When several different species were individuated in a spectrum, the same FWHM value was used for all of the individual photoemission bands [77].

Atomic Force Microscopy (AFM) measurements have been performed in both air and fluid, using a Veeco Multimode™ model (New York, NY, USA) equipped with a Nanoscope IIIa controller. Measurements in air have been performed in tapping mode, to acquire topography, amplitude, and phase data. The samples of nanoparticles were prepared by the deposition of a drop of water on a wafer of clean Si/SiO_2_ with a negligible roughness, with respect to the typical dimensions of the particles. On the other hand, the membranes were directly stacked on the sample holder, using an epoxy resin. In fluid, measurements were collected using a Veeco glass fluid cell filled with a suitable aqueous solution, both immediately after filling, and with increasing time. Moreover, measurements in air were carried out on membranes that had previously stayed in contact with the solution. In this case, a drop of water was deposited on the membranes stacked on the holder. After a fixed time, the water was removed, the membranes were dried, and the measurement was carried out in air, as already described. Images were recorded with a 512 × 512 pixels resolution and corrected by polynomial background filters using the Gwyddion 2.31 software. FESEM measurements have been carried out on a SiO_2_ substrate with a Auriga Zeiss instrument (resolution 1 nm, applied voltage 6–12 kV, Oberkochen, Baden-Württemberg, Germany), on freshly deposited membranes without metallization.

A Mini Spin—Eppendorf centrifuge was used for the purification of AuNP samples (13,000 rpm), and pH measurements were made with a CyberScan pH 600 (Eutech Instruments, Thermo Scientific™, Landsmeer, The Netherlands). Deionized water was obtained from Zeener Power I Scholar-UV (18.2 MΩ, Human™, Soul, Korea).

## 4. Conclusions

In this work, the preparation of a nanocomposite made from sPEEK membranes and gold nanoparticles, through an in situ synthesis protocol by precursor impregnation in the host membrane, is reported. Prior to impregnation, the pristine sPEEK membrane was nanostructured (nanophase separation between its hydrophilic and hydrophobic domains) by an ad-hoc hydrothermal treatment, allowing us to control the morphology of the host membrane and therefore, of the nanocomposite membrane. The reduction of HAuCl_4_ in the presence of the reducing agent NaBH_4_, using 3MPS thiols as a stabilizing ligand, was carried out in the presence of the sPEEK membrane. The composite membranes have been characterized by FTIR, UV-vis, XPS, FESEM, and AFM. The characterizations showed the presence of AuNPs with a diameter smaller than 10 nm; although, in the latter case, the size could not be determined. In particular, AFM measurements in fluid were also performed, to study the behavior of the membrane in conditions of hydration, which showed a strong tendency of the membrane to swell. To conclude, the procedure has led to the preparation of composites based on sPEEK, containing gold nanoparticles, which are potentially useful in fuel cell applications.

## Supplementary Materials

The following are available online at www.mdpi.com/1996-1944/10/3/258/s1. Figure S1: FTIR spectra of the sPEEK membrane (green line), Au-3MPS NPs (blue line), nanocomposite Au-3MPS@sPEEK (red line); and free 3MPS thiol (brown line). Table S1: XPS data.
