# Supplementary material for: Nanocomposite Based on Functionalized Gold Nanoparticles and Sulfonated Poly(ether ether ketone) Membranes: Synthesis and Characterization
Source: Materials (Basel). 2017 Mar 3;10(3):258. doi: 10.3390/ma10030258 (PMC5503356; doi:10.3390/ma10030258)
Supplement: Supplementary file 1 [file materials-10-00258-s001.pdf]

Supporting Information

Nanocomposite based on functionalized gold nanoparticles and sulfonated poly(ether ether ketone) membranes: synthesis and characterization

Iole Venditti[1], Laura Fontana[1], Francesca A. Scaramuzzo[2], Maria Vittoria Russo[1], Chiara Battocchio[3], Laura Carlini[3], Laurent Gonon[4], Vincent H. Mareau[4], Ilaria Fratoddi[1]*

[1] Department of Chemistry, Sapienza University of Rome, P.le A. Moro 5, 00185 Rome (Italy)Roma Tre University, Dept. of Sciences, Via della Vasca Navale 79, 00146 Italy

[2] Department of Basic and Applied Sciences for Engineering, Sapienza University of Rome, Via A. Scarpa 14, 00161 Rome (Italy)Sapienza University, Dept. of Chemistry, P.le A. Moro 5, 00085 Rome Italy

[3] Dept. of Sciences and CISDiC, Roma Tre University, Via della Vasca Navale 79, 00146 - Rome (Italy)

[4] Univ. Grenoble Alpes, CNRS, CEA, INAC-SPrAM, F-38000 Grenoble, France

* Correspondence: ilaria.fratoddi@uniroma1.it

Figure SI-1: FTIR spectra of the sPEEK membrane (green line), Au-3MPS NPs (blue line), nanocomposite Au-3MPS@sPEEK (red line); and free 3MPS thiol (brown line).

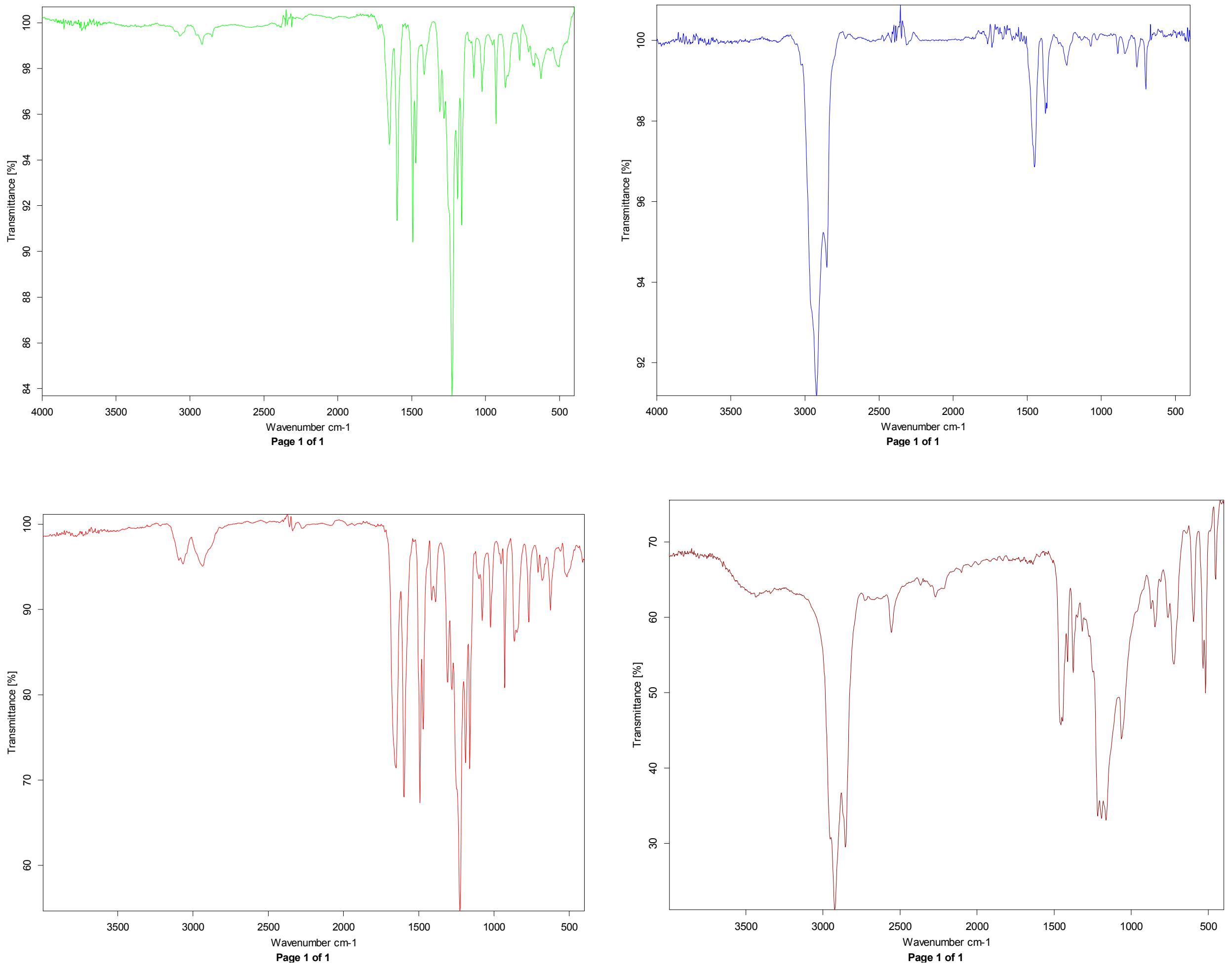

Table 1: XPS data.

sPEEK	Signal	BE (eV)	FWHM (eV)	Relative Intensity (I_{signal}/σ)	Assignment
	C1s	285.00	1.68	7364.1	C-C
		286.56	1.68	1883.9	C-S, C-O
		288.12	1.68	884.3	C=O
		289.32	1.68	489.2	COOH
	$S2p_{3/2}$	163.13	1.93	22.2	-SR
		167.24	1.93	30.2	$-SO_3^-$
	$Au4f_{7/2}$	83.98	0.98	0.6	Au (0)
		84.82	0.98	0.35	Au (δ^+)
	B1s	190.43	3.25	153.8	BO_3^{3-}
	$Cl2p_{3/2}$	198.27	2.72	71.2	NaCl
	O1s	530.06	1.97	791.3	Inorganic O
		532.09	1.97	2338.4	Organic O
		533.54	1.97	659.7	Physisorbed H_2O
